# Tracking supercritical geothermal fluid distribution from continuous seismic monitoring

**DOI:** 10.1038/s41598-023-35159-8

**Published:** 2023-05-24

**Authors:** Rezkia Dewi Andajani, Takeshi Tsuji, Tatsunori Ikeda, Satoshi Matsumoto, Keigo Kitamura, Jun Nishijima

**Affiliations:** 1 School of Engineering, The University of Tokyo, 731 Hongo, Bunkyo-ku, Tokyo, 113-8656 Japan; 2 Department of Earth Resources Engineering, Kyushu University, 744 Motooka, Nishi-ku, Fukuoka, 819-0395 Japan; 3 International Institute for Carbon-Neutral Energy Research (WPI-I2CNER), Kyushu University, 744 Motooka, Nishi-ku, Fukuoka, 819-0395 Japan; 4 Institute of Seismology and Volcanology, Kyushu University, 2‐5643‐29, Shin’yama, Shimabara, Nagasaki 855‐0843 Japan

**Keywords:** Geophysics, Volcanology

## Abstract

Continuous seismic monitoring could play a pivotal role in deep geothermal energy exploration. We monitored seismicity near geothermal production areas of the Kuju volcanic complex with a dense seismic network and automated event detection. Most events were shallow (less than 3 km below sea level) and distributed along a boundary between regions of high and low resistivity and S-wave velocity, interpreted as a lithological boundary or related fracture zone. Deeper events located on top of subvertical conductors may reflect fracturing associated with magmatic fluid intrusion. A correlation may exist between seismicity and heavy rainfall three days prior to increased pore pressure in pre-existing fractures. Our findings support the presence of supercritical geothermal fluids and demonstrate the importance of continuous seismic monitoring in supercritical geothermal energy exploration.

## Introduction

The need to achieve carbon neutrality by 2050 has encouraged exploration for deep supercritical geothermal energy sources. Because of their exceptionally high temperature and enthalpy, supercritical geothermal fluids (T > 374 °C and P > 22.1 MPa for pure water, T > 406 °C and P > 29.8 MPa for seawater)^1–4^ may offer more energy than conventional geothermal systems. Deep supercritical geothermal fluid is often located at depths near the brittle–ductile transition (BDT)^1–4^ where an impermeable sealing horizon allows pressures to exceed hydrostatic pressure^5^. Supercritical geothermal fluid is often connected to active volcanoes^6^. Given the inferred high pressure and temperature, the rocks in this environment likely exhibit ductile behavior. It has been proposed that supercritical geothermal fluid presents a relatively low risk of brittle failure, which triggers seismicity in geothermal energy developments^7,8^.

In the vicinity of active volcanoes, supercritical fluids could be present at depths shallower than 10 km. Drilling experiments have encountered geothermal fluids at suitably high temperatures at depths about 2–5 km^2,3,9–11^. In Japan, supercritical geothermal fluid may exist in several regions, such as Kakkonda in northeastern Japan^12,13^ and the Kuju volcanic complex in northern Kyushu, southwestern Japan^14,15^. In the Kakkonda field, various studies implemented seismic and magnetotelluric survey^16,17^ have suggested that supercritical geothermal fluids may be present in the core of a relatively aseismic, low-resistivity zone beneath depths of 3 km below sea level with a steep temperature gradient from ~ 380 to 500 °C.

In the Kuju volcanic complex, geophysical field surveys have been conducted to characterize the deep geothermal reservoir^18–22^, but the connection between the distribution of geothermal fluids and the BDT is still unclear. In this case, earthquakes are often located near the BDT^3,23,24^. Rocks near the BDT can be brittle in extension^25^ and capable of generating cracks. If such cracks could be detected through microseismic monitoring, it could help identify the position of the BDT. In this study, we sought to characterize the BDT as well as fluid pathways in Otake-Hatchobaru geothermal field at Kuju volcano from the distribution of earthquakes detected by a dense local seismic network.

We compared our result with the previous study of resistivity^21^ and shallow S-wave velocity model within surface to 2 km beneath sea level^20^. Earthquakes in our result mostly clustered around the Otake and Hatchobaru geothermal power stations, and their locations favored the boundary between areas of high and low Vs and resistivity values. We interpret this trend as the preferential formation of cracks in relatively brittle areas where fluid pathways may follow lithological boundaries. Shallow seismicity (1–2.7 km below sea level) may reflect hydrothermal fluid activity along the fault systems in the geothermal area, whereas deeper earthquakes (> 3 km) may reflect crack generation at the boundary of the brittle-ductile transition. We further evaluated the possibility of rainfall triggering seismic activity in volcanic regions. We concluded that seismicity may be triggered when surface loading due to rainfall infiltration coincides with hydrothermal fluid circulation within the fault network as under critically stressed conditions, even small variations in pore pressure can generate cracks that increase fault permeability.

## Result

### Study area

**Figure 1:**
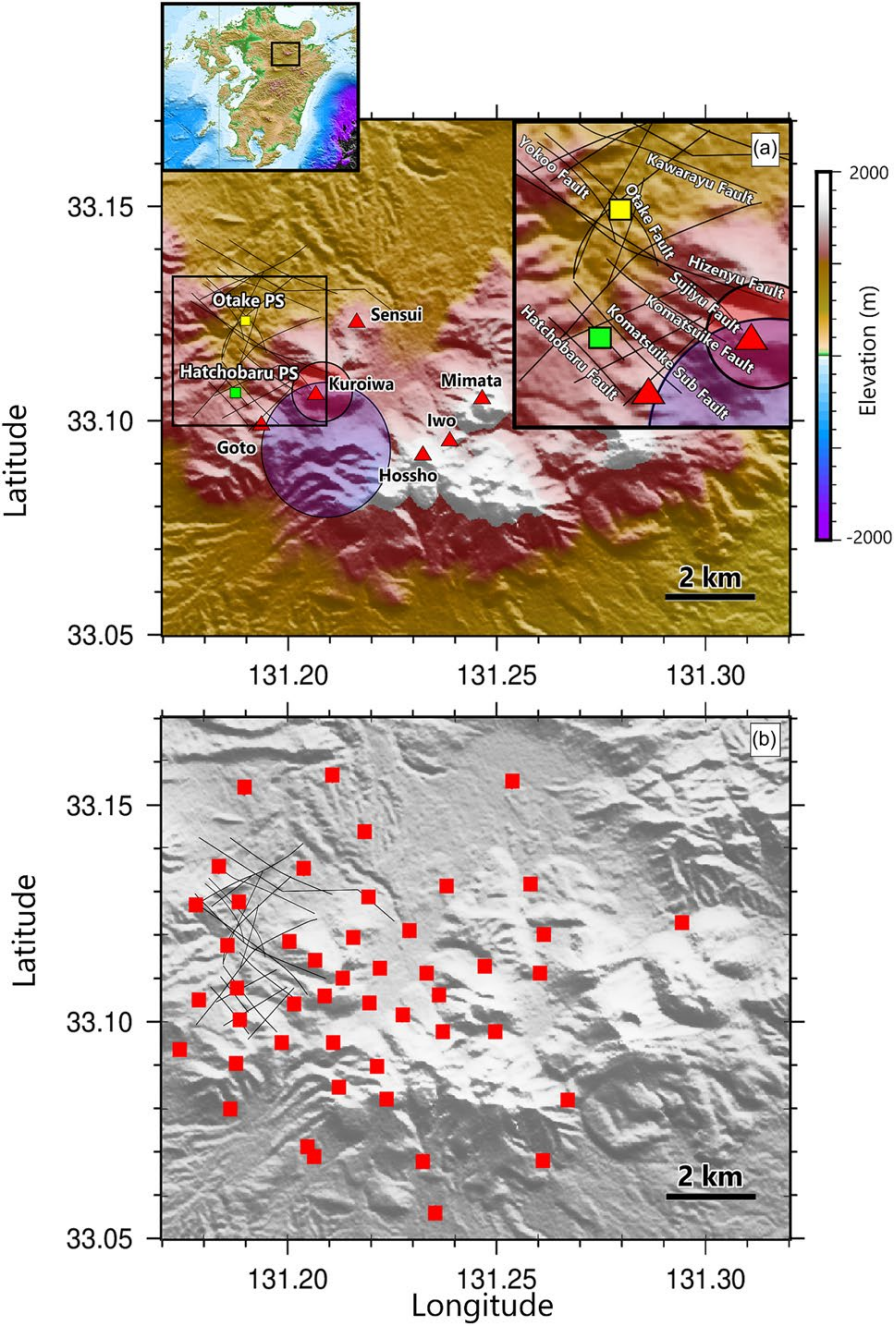
Description of our study area. (**a**) Location map showing the topography of the Kuju volcanoes (red triangles) and the location of the Otake and Hatchobaru power stations (PS). The black lines around the power stations are faults^14,18^. The blue shaded circle indicates the interpreted heat source at 5 km below sea level^21^. The red shaded circle shows the interpreted body of geothermal fluid beneath Mt. Kuroiwa^14^. The inset shows the location of the study area on Kyushu Island. (**b**) Seismometer locations in the study area marked by red rectangular symbol.

Starting 21 May 2022, we deployed a network of 47 seismometers around the Kuju volcanoes (Fig. 1). The instruments were vertical-component seismometers with a sampling frequency of 100 Hz. The seismometer network was deployed at an altitude of roughly 1 km above sea level and surrounded the young volcanoes (< 0.2 Ma) including Mt. Goto, Mt. Sensui, Mt. Kuroiwa, and Mt. Iwo (Kuju-Iwoyama). The Otake and Hatchobaru geothermal power stations are located about 5 km northwest of Mt. Iwo. The geological structure in that area is characterized by the Beppu–Shimabara graben, associated with volcanic rocks of Miocene to Pleistocene age^26^. There the Kuju volcanic rocks at the surface are underlain successively by the Hohi volcanic group, the Kusu and Usa groups, and granitic basement. The Otake-Hatchobaru geothermal system is primarily governed by a network of NW- and SE-trending fractures (Fig. 1a). The faults in this area are confirmed at elevation − 500 m (500 m below sea level) by drilling (e.g., Hatchobaru, Komatsuike, Sujiyu, Otake, Yokoo, and Kawarayu fault) and geophyisical survey (e.g., Hizenyu fault)^14^. The heat source is speculated to lie beneath Mt. Kuroiwa, Mt. Sensui, and Mt. Goto^14,27–29^. The geothermal reservoir is formed by heated meteoric water that migrates through the fault system^14^. In the Otake area, the fluid migrates along the Sujiyu and Hizenyu faults, then flows upward through the Yokoo and Otake faults, forming a reservoir beneath an altered zone 200–500 m below the surface^14,29^. In the Hatchobaru area, the heat source is believed to be from Mt. Goto^27^, and the fluid migrates through fractures along the Hatchobaru fault and the Komatsuike subfault^28^.

**Figure 2:**
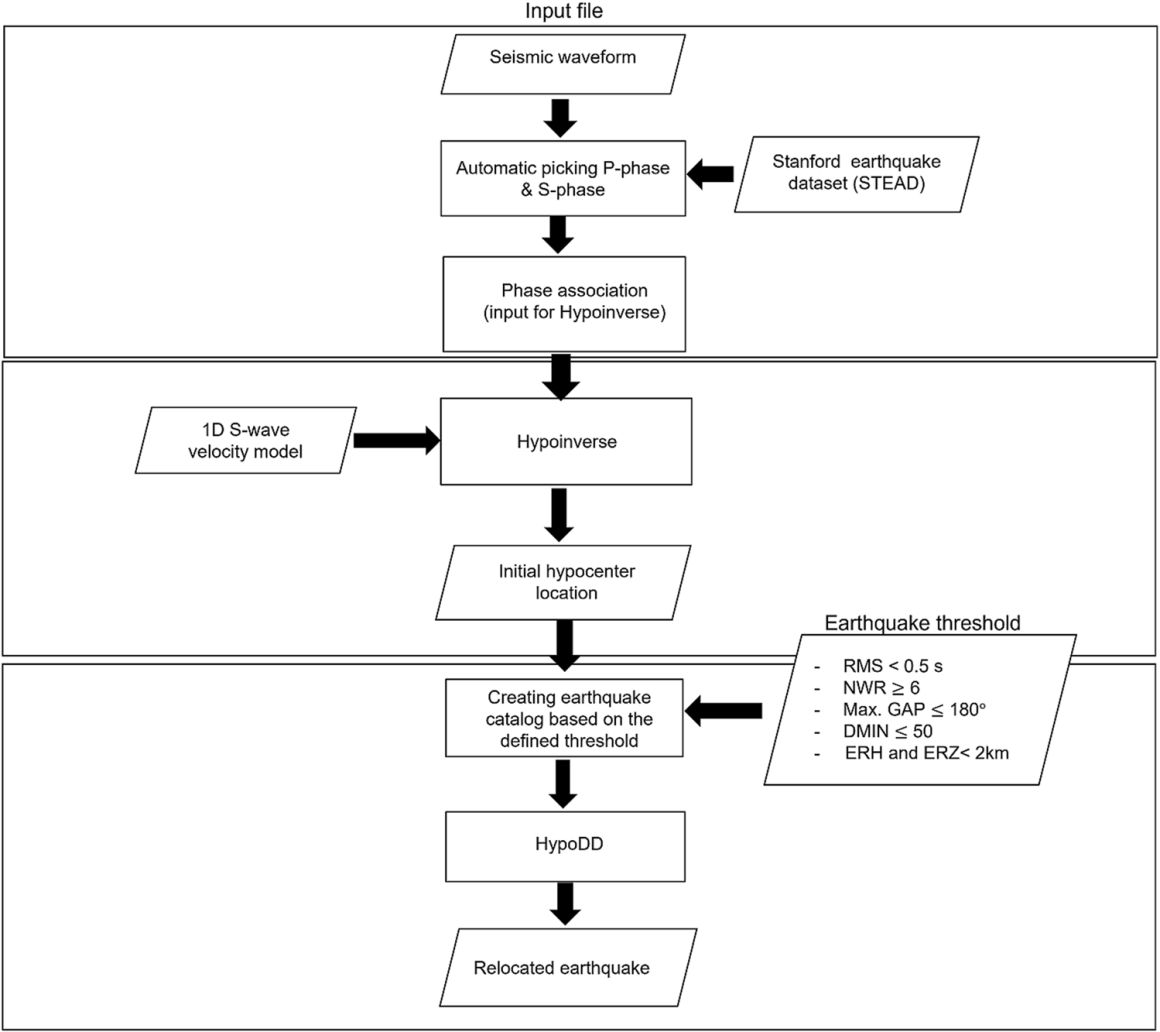
Flowchart summarizing the method in this study.

To estimate hypocenter distributions from the seismic data, we used a three-step approach (Fig. 2). First, we used the EQTransformer (EqT) program^30^ to automate the seismic event detections. We estimated the hypocenters of these events with the Hypoinverse-2000 program^31^, then relocated the earthquake by the double difference method (HypoDD)^32^. Prior HypoDD, we selected the earthquake based on the factors that govern the precision of estimated hypocenters: RMS (root-mean-square residual), DMIN (epicenter distance to the nearest station), GAP (the largest azimuthal gap between nearby stations, measured from the epicenter), NWR [number of weighted station readings (phases)], ERH (horizontal location error), and ERZ (vertical location error).

### Earthquake identification

**Figure 3:**
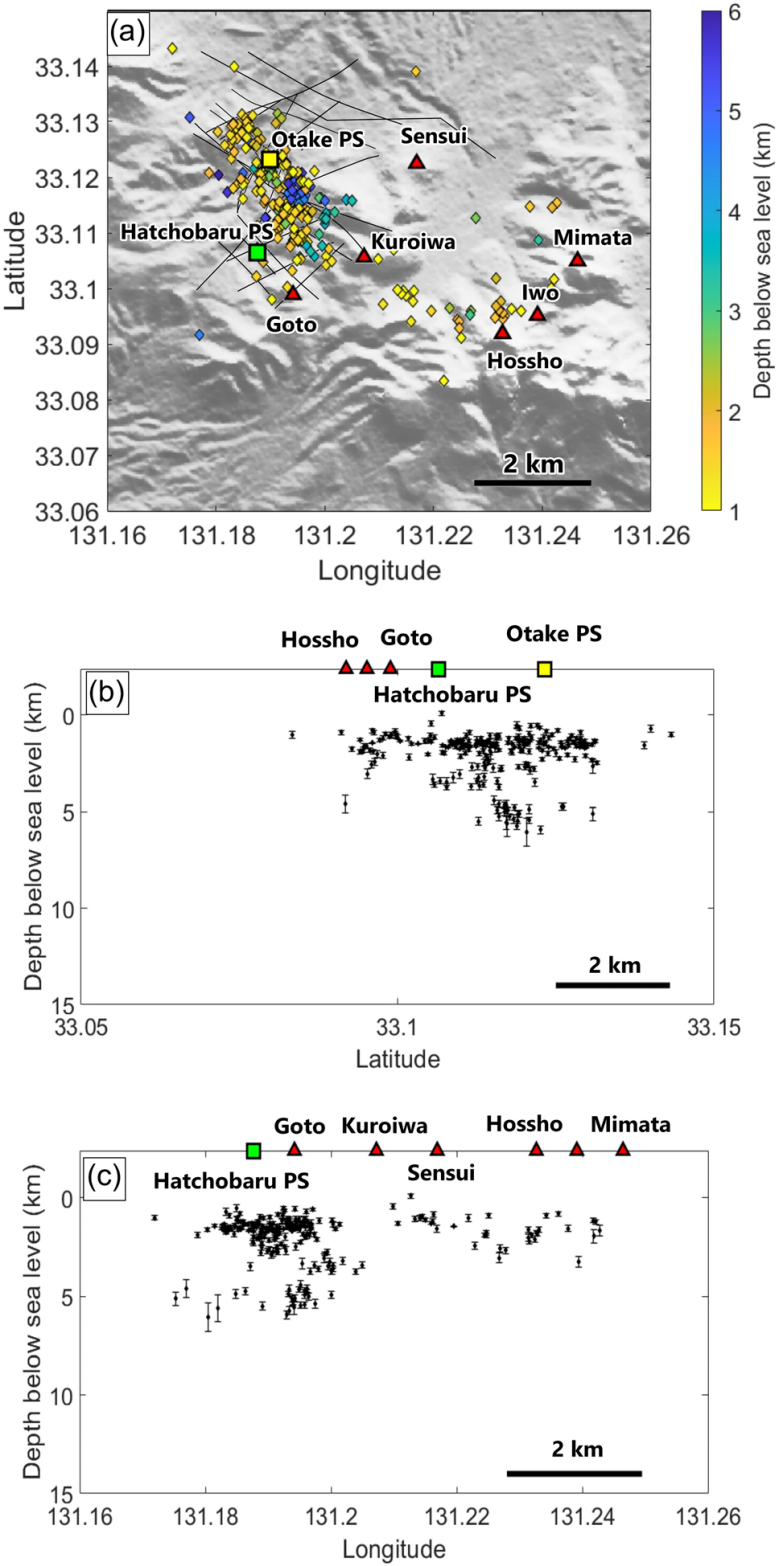
Hypocenter results after relocation by HypoDD. (**a**) Plan view of hypocenters, with depths represented by color. (**b**) North–south and (**c**) east–west vertical profiles of hypocenters, with its vertical error represented.

From 2600 seismic events automatically detected during the 21 May to 27 July study period, ~ 620 events were identified close to Kuju volcanic complex, and 259 earthquakes satisfied our threshold criteria. Most earthquakes were concentrated around the Otake and Hatchobaru power stations (Supplementary Fig. 1) and had the following values: RMS = ~ 0.1 s, horizontal location error = 0.13–1.43 km with an average of 400 m, and vertical location error = 0.1–1.8 km with an average of ~ 500 m (Supplementary Figs. 2 and 3). Of the 259 events, 250 were relocated by HypoDD, reducing the vertical location error to 19–708 m, with an average of 119 m (Fig. 3).

The plan view (Fig. 3a) shows that most seismicity was distributed in a N–S trend beneath the two geothermal power plants. Projected vertical profiles showed that the events formed two clusters at depth ranges of 1–3.5 km and 4–6 km below sea level, with most hypocenters clustered within 1–2 km depth (Fig. 3b,c). Compared to the events before relocation (Supplementary Fig. 2), the clusters of the earthquake after relocation were slightly shifted towards north, closer to Komatsuike and Sujiyu fault where geothermal fluids could migrate through^14^.

## Discussion

**Figure 4:**
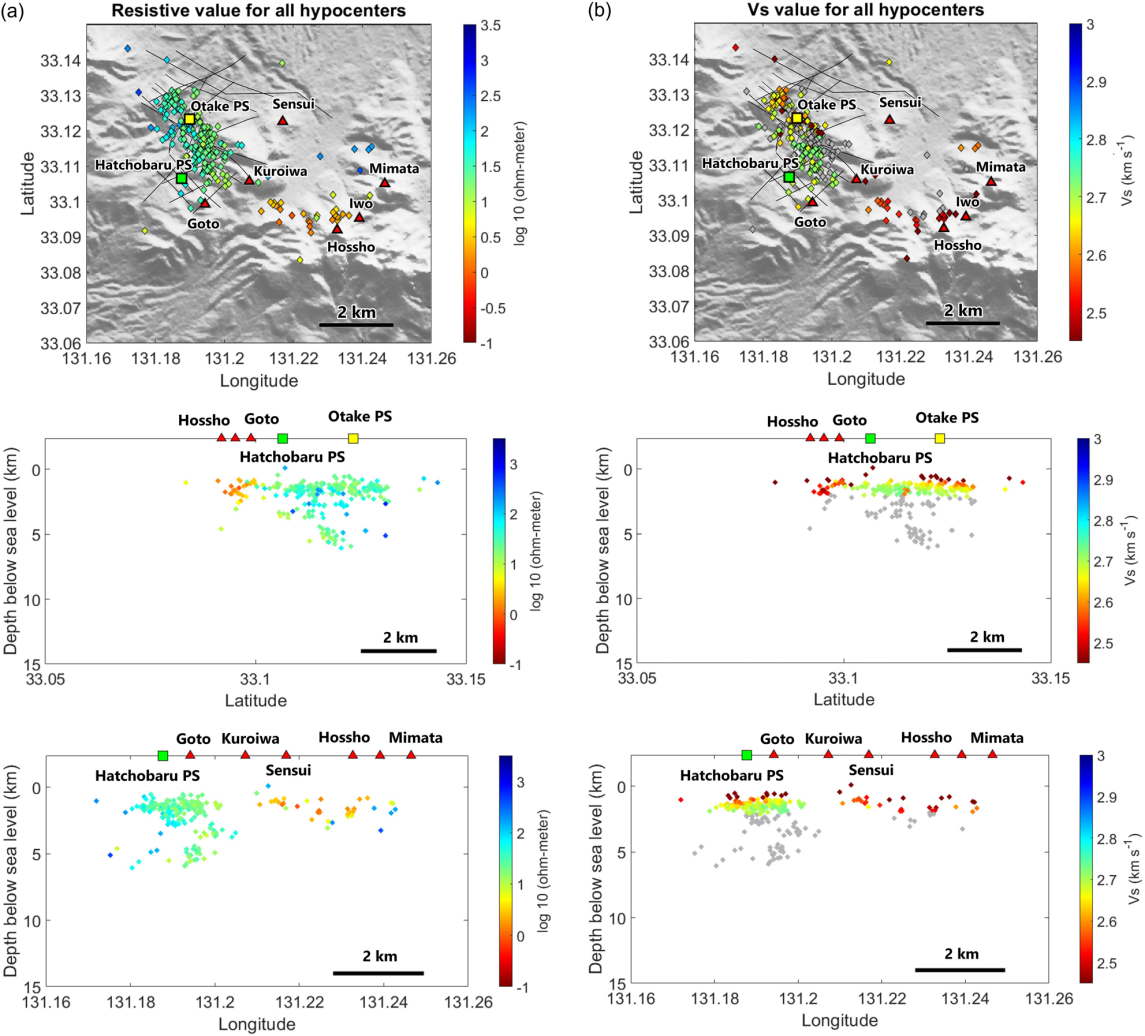
Maps showing inferred approximate values of (**a**) resistivity^21^ and (**b**) Vs ^20^ at hypocenter locations. Hypocenters below 2.7 km depth where there is no information of Vs value, are shown in gray.

**Figure 5:**
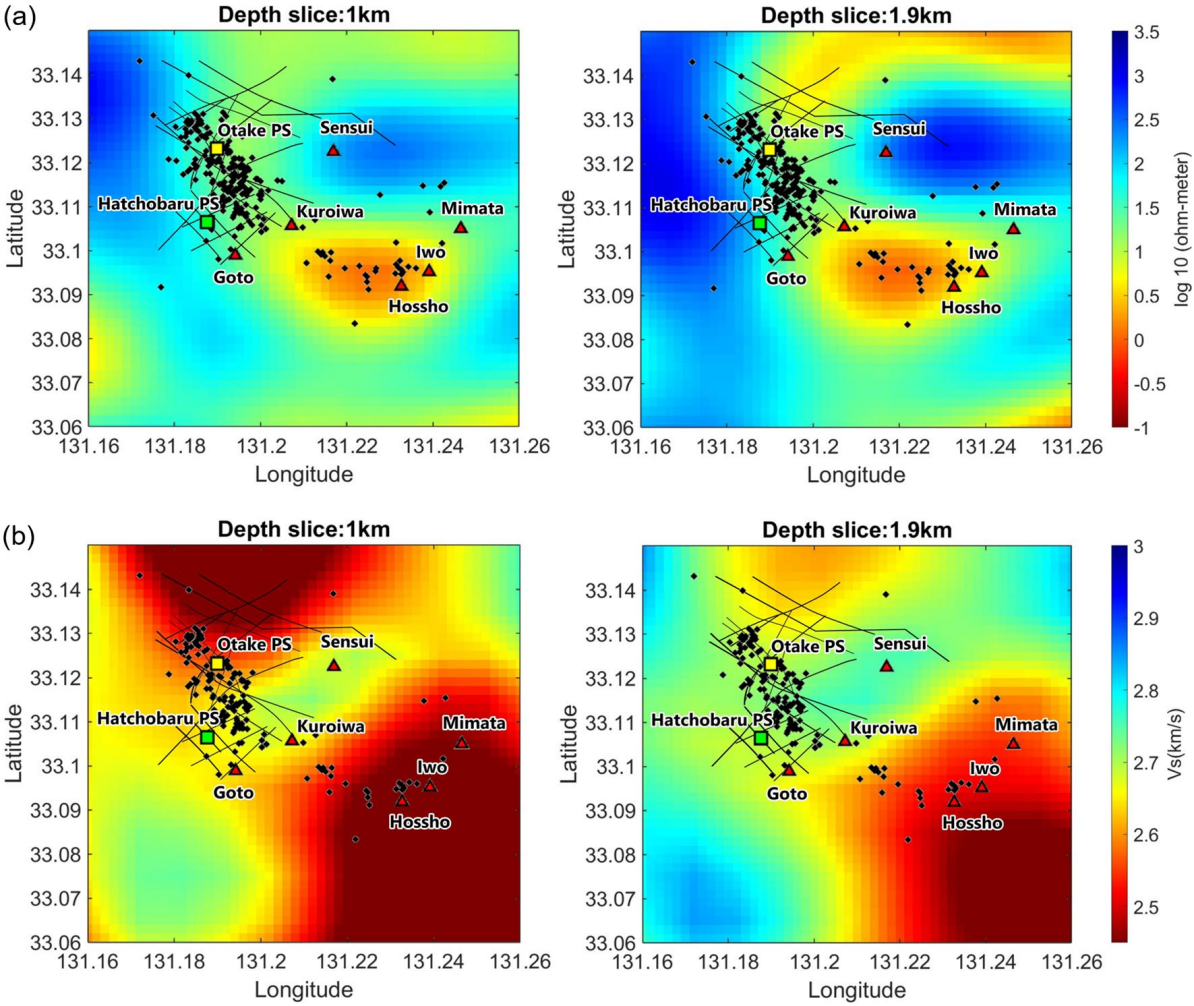
Horizontal slices showing interpolated values of (**a**) resistivity^21^ and (**b**) Vs^20^ at 1.0 and 1.9 km depth with shallow hypocenters superimposed. Panel (**a**) shows the hypocenters for all depths, while in panel (**b**) hypocenters are displayed within 1–1.9 km depth below sea level.

**Figure 6:**
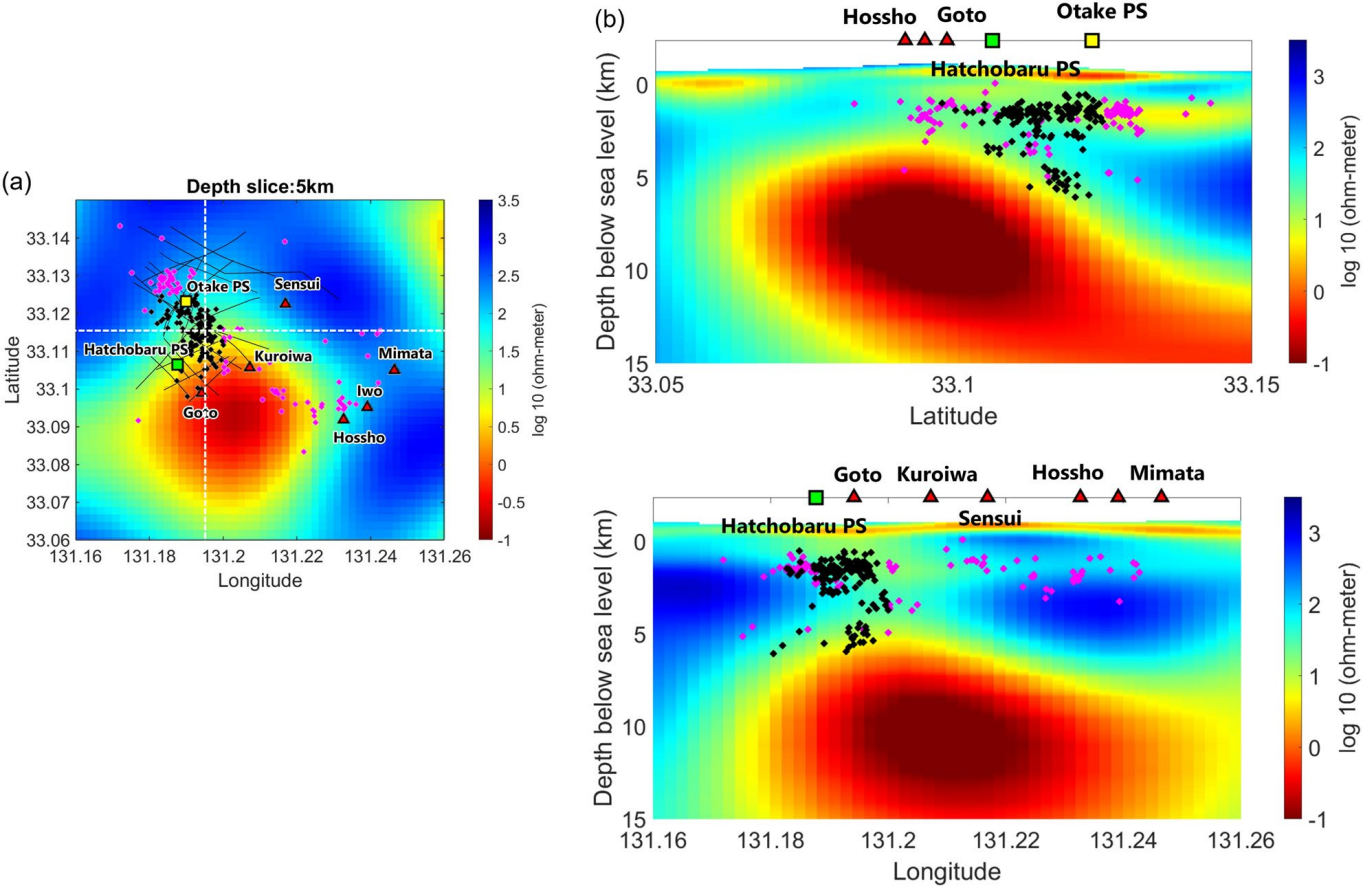
Horizontal slice showing (**a**) the plain view of resistivity (~ 5 km depth below sea level^21^) and (**b**) vertical slice of both south–north and east–west resistivity profiles. The seismicity marked with magenta color represents the cluster of the seismicity which is farther from the MT slice profile we used.

Figure 4a shows the approximate resistivity values at each hypocenter. The resistivity associated with the hypocenter is centered around 25 Ω-meters (1.4 on the log 10 scale) and is lower to the southeast of power stations (extending to Mt. Hossho). Figure 4b shows that Vs values at most of the shallow hypocenters are about 2.5–2.7 km s^−1^, with lower values (1.9–2.4 km s^−1^) found southeast of the power stations. The less intense seismicity in the southern part of the cluster, where resistivity and Vs are lower, signifies a less brittle area. Overlaying our results with the resistivity^21^ and S-wave velocity profiles^20^ (Figs. 5 and 6) shows that the hypocenters are mostly clustered near boundaries between lower and higher values of resistivity and Vs. This result is consistent with previous studies finding that earthquakes are often observed in the vicinity of resistive and conductive zones, specifically within more-resistive rocks^33–35^. This suggests that geothermal fluids migrate along lithological boundaries, which commonly concentrate stress and develop fractures or cracks that favor fluid migration.

The earthquakes occupied an oval region aligned N–S within a depth range of 1–6 km below sea level. The seismic clusters shallower than 2 km appear to be associated with the hydrothermal system (Fig. 5). In the area of the power stations, heated meteoric water circulates above the granitic basement at a depth of 700 m below sea level^36^. The relative paucity of hypocenters to the east of the power stations, where resistivity and Vs are lower, suggests the presence of a heat source in that area. The meteoric water is heated southeast of the power stations and then migrates northwest along fracture networks^14,28^. Fluid migration along the faults in this area may generate cracks through increases in pore pressure, thus enhancing fault permeability^34,37,38^. Given the agreement of our interpretation and previous ones, we interpret the earthquakes as cracking events associated with geothermal fluid migration.

Seismic clusters at depths greater than 3 km may reflect crack generation related to an apparent subvertical conduit extending from Shishimuta caldera^21^ toward Mt. Kuroiwa and Mt. Goto (Fig. 6). Repeated magma intrusion below a volcanic complex generates a large volume of plastic rocks at high temperatures (> 400 °C) that result in a BDT^5^. The sealing zone near the BDT episodically breaches, allowing magmatic fluid to discharge into shallower hydrothermal systems^5^. The edge of this conduit could be highly permeable and constitute a fluid pathway^21^. Although permeability near the BDT has been proposed to decrease drastically with increasing temperature^39^, laboratory experiment support the absence of a sharp permeability decrease near the BDT^4^. It is implied that magma intrusions could cause hydraulic fracturing that increases permeability at elevated temperature (e.g., 375–460 °C at 2–6 km depths)^4,25^, hence it is possible for some zones of high permeability to exist near the BDT. Previous studies have suggested that heat sources with temperatures in the 400–700 °C range are present at 5 km depth near Mt. Iwo and Mt. Kuroiwa^14,37^. The temperature profile east of the Otake-Hatchobaru geothermal area is interpreted to reach 380 °C at about 3 km below sea level, as indicated by the record from a well close to the geothermal area^22^. Hence, the configuration of earthquakes and their proximity to the sub-vertical conduit are consistent with crack generation due to fluid migration from a heat source toward shallower hydrothermal systems. Given the temperature range of the heat source and the distribution of seismicity, there is a possibility that a supercritical geothermal resource exists east of the Otake-Hatchobaru area.

**Figure 7:**
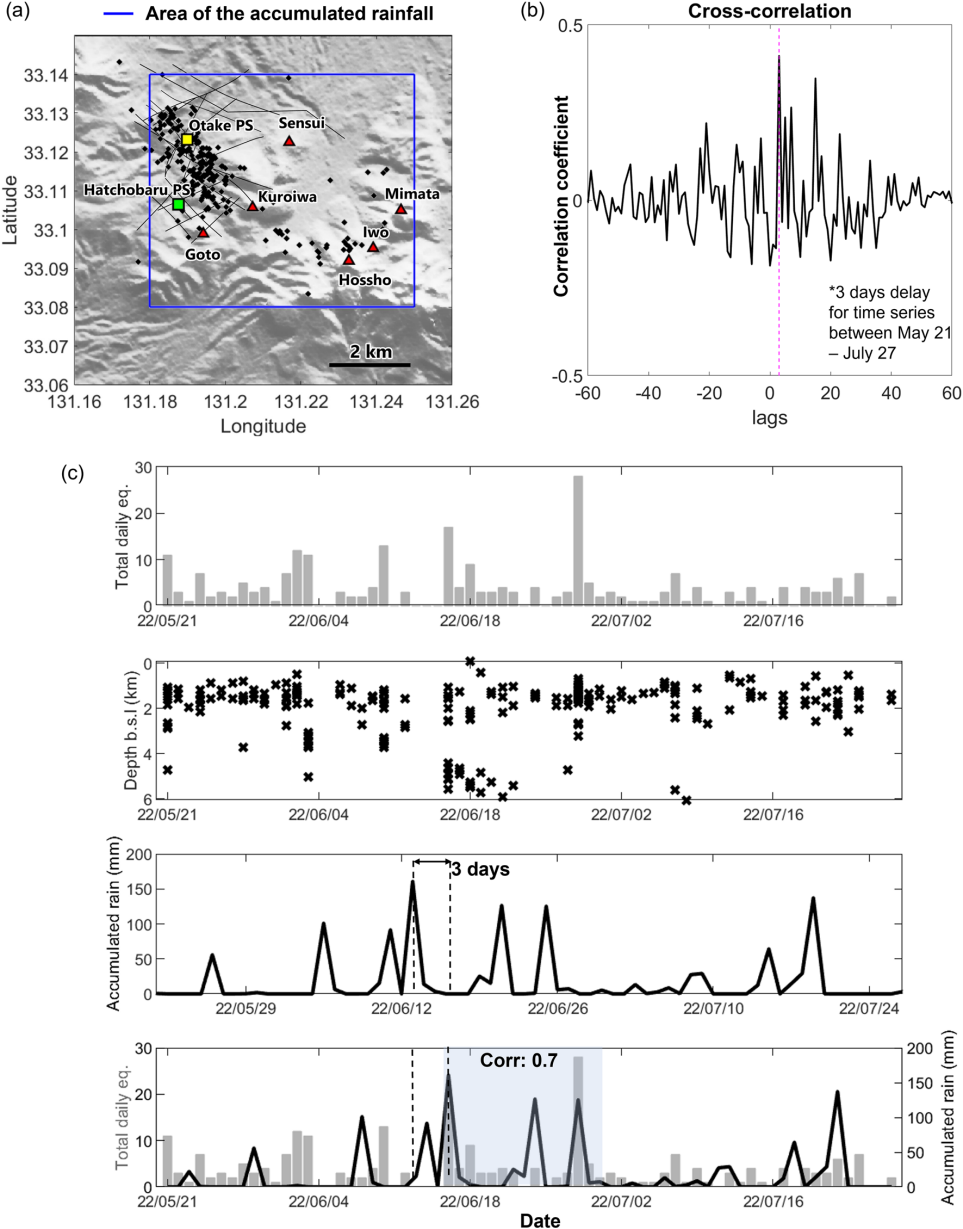
Comparison of earthquake and rainfall data. (**a**) Map of the study area showing the area (blue rectangle) in which the accumulated rain was calculated from Global Satellite Mapping of Precipitation (GSMaP) data^43^. (**b**) Time domain cross correlation between rainfall and subsequent earthquakes. (**c**) Plot of daily total earthquakes, hypocentral depths, accumulated rainfall, and a comparison of earthquakes and rainfall 3 days earlier (correlation coefficient was calculated for the shaded region).

Various studies have investigated the likelihood of rainfall triggering seismic activity in volcanic regions^40–42^, and the geothermal fluids in Otake-Hatchobaru area are primarily sourced from meteoric water^14^. A longer observation period is needed to investigate the relationship between rain precipitation and earthquake intensity, and our observation period seems to be short for such an analysis. However, previous study^42^ demonstrated a clear relationship between rainfall and pore pressure change that could trigger cracks opening in volcanic region. We therefore modeled the possibility that the surface load from rainwater infiltration triggered the inferred cracking (seismicity) in the 1–6 km depth range in our study area. Figure 7 compares the record of daily total earthquakes in our observations with accumulated rainfall in the study area. The highest seismicity occurred on 16 and 28 June whereas the greatest daily rainfall occurred on 13 June (160 mm) and 25 June (125 mm). Considering that there would be a delay for rainwater infiltration to trigger earthquakes, we evaluated a 3-day delay of rainfall with respect to the seismicity time series (Fig. 7b) and obtained a correlation coefficient of 0.4 for the whole time series and 0.7 for the time series between 15 and 30 June. Indeed, in our results, there seems to be relationship between rain precipitation and earthquake. Thus, the seismicity peaks on 16 and 28 June could coincide with peaks in pore pressure as surface loading combined with geothermal fluid migration.

Overburden pressure imposed by a rise in groundwater could influence the underlying stress state and pore pressure^42,44^, and under critical conditions, even slight variations in pore pressure could trigger cracking that increases the permeability of pre-existing fractures. The passage of hydrothermal fluid through the fracture network would decrease the pore pressure and result in a decrease in seismicity. Note that earthquakes are not solely affected by the overburden from rainfall. Hydrothermal fluid activity could alter the pre-existing fracture network and explain the earthquakes that occurred around 1–3 June, when rainfall was relatively low. We conclude that the occurrence of earthquakes reflects the migration of hydrothermal fluid along a lithological boundary. This finding may help further identify the location of supercritical geothermal resources around the Kuju volcanoes.

## Methods

### Seismic event detection

We used EqT to detect P- and S-wave arrival times^30^. The seismic waveform was sliced into 1-min windows that overlapped in time by 50%. The program automatically detects seismic events based on deep learning. The model in the program was pre-trained on the Stanford Earthquake Dataset (STEAD)^45^. The waveforms in STEAD are mostly from earthquakes from diverse locations with epicentral distances < 100 km and hypocenter depths shallower than 50 km, making it suitable for local earthquakes^46,47^.

**Figure 8:**
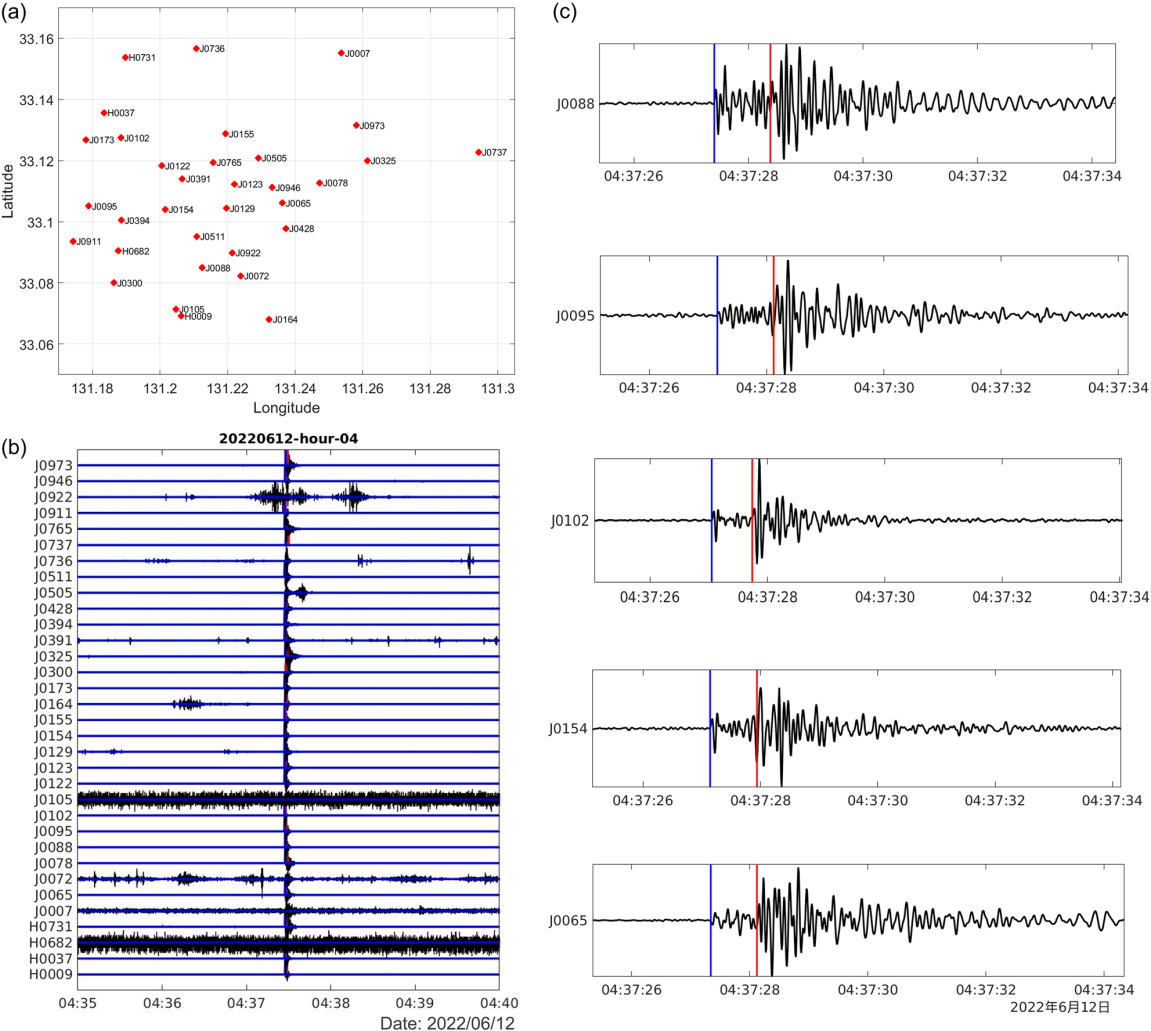
An example of automatic detection result on June 12th, 2022, 4 AM Japan Standard Time (JST). (**a**) Seismometer locations and (**b**) corresponding waveforms filtered within 1–45 Hz. (**c**) Examples of detected P-wave (blue) and S-wave (red) arrivals at five stations.

The performance of EqT is influenced by its assigned threshold values of probability for event detection^30^. In this case, we used threshold values of 0.3 for event detection and 0.1 for P- and S-wave detections. The use of a pre-trained model that is robust against false positives allowed us to use a relatively low threshold value^30^. EqT detected seismic events independently for each seismic station (Fig. 8). During the phase association step, similar events had to be detected at a minimum of three stations to constitute the occurrence of an earthquake. The output from this process can be used directly in the Hypoinverse program. Before this step, we confirmed that the detected events were consistent with those identified manually.

### Velocity model for earthquake determination

**Figure 9:**
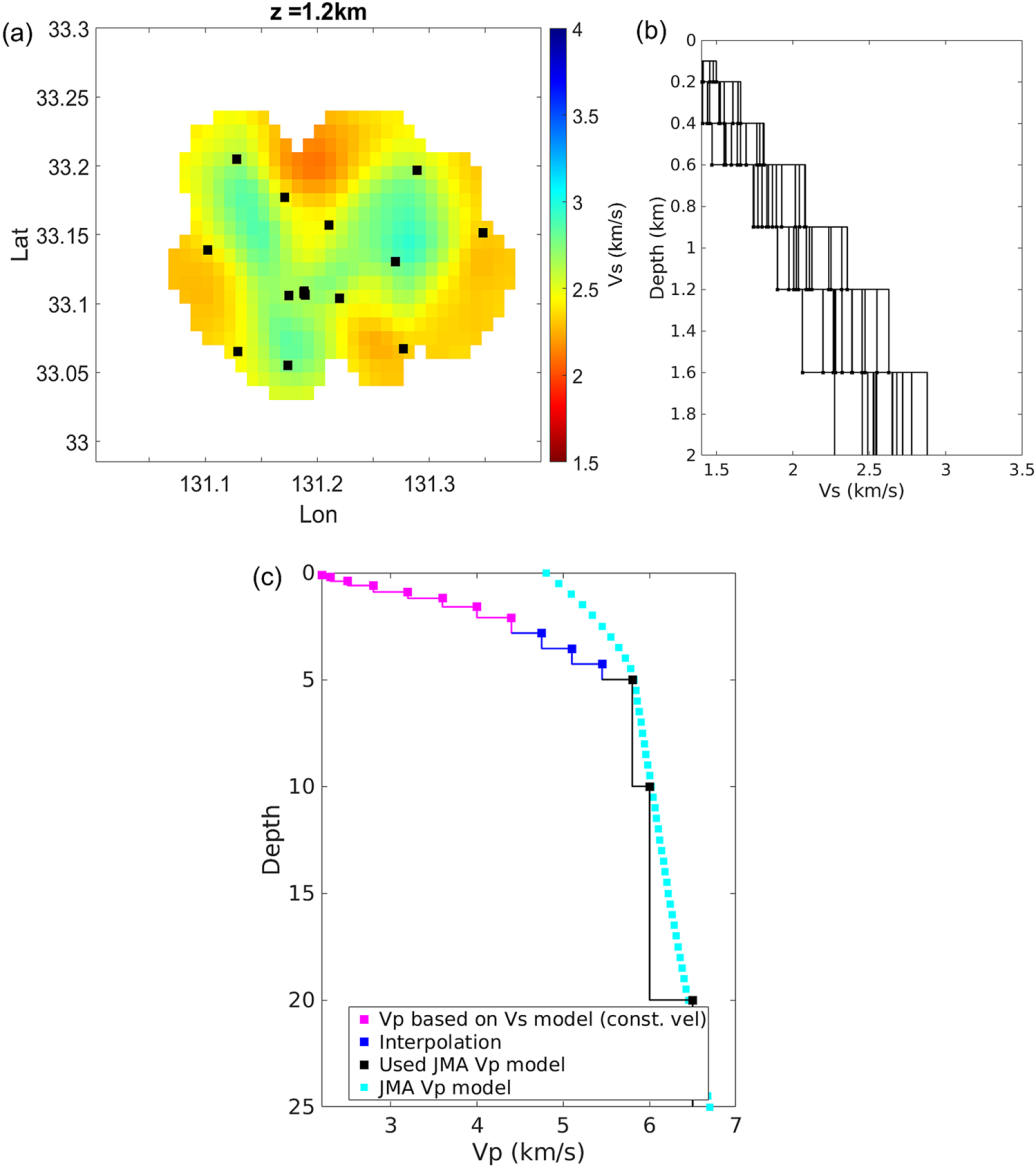
(**a**) Map of seismic stations and displayed S-wave velocity model^20^ for a depth of 1.2 km from the surface. The rectangular symbol in the S-wave velocity layer represents the station location in the previous study^20^. (**b**) Depth profile of S-wave velocities for the stations in panel (**a**). (**c**) The final P-wave velocity model used for hypocenter estimation. Velocities above 2 km depth were obtained by averaging all velocities in panel (**b**). The 1-D JMA velocity model^53^ was used for depths greater than 5 km (marked by black colour).

For determining earthquake locations, we used an S-wave velocity (Vs) model for the study area previously estimated by our group (Fig. 9)^20^. This velocity model was constructed by ambient noise tomography using the zero-crossing method^48^, which estimates phase velocity based on a spatial autocorrelation method. Then, we applied a direct surface wave inversion method^49^. This approach has been used in various studies to resolve shallow crustal features^50–52^. Prior to the zero-crossing step, the daily seismic waveform was divided into 30-min segments with 50% overlaps and bandpass filtered between 0.2 and 0.7 Hz. Station pairs were established between all 18 seismometers (Fig. 9), and power normalized cross-correlation spectra were then derived for each station pair. The phase velocities (zero crosses) for each station pair were calculated from the stacked cross-correlation spectra from all possible data periods. Finally, the phase velocity dispersion curve from the zero-crossing method was determined based on the velocity of the 0.2 Hz reference phase. The 3D S-wave velocity structure for each station pair was estimated through direct surface wave tomography^49^.

To obtain 1D velocity models for hypocenter determinations, we took the velocity models for each station location and horizontally averaged them. The Vp/Vs ratio in northwestern Kuju is less than 1.73 for depths greater than 2 km below sea level and ranges from 1.65 to 1.85 at shallower depths^54^. We assumed a Vp/Vs ratio of 1.7 to convert our Vs model to a Vp model. Because our Vs model was sensitive to local heterogeneity at shallow depths (< 3 km), we combined our 1D velocity model at the shallower depth with the JMA velocity model^53^ for depths below 5 km and interpolated between the two models at intermediate depths. Figure 9c shows the resulting velocity model used to estimate event locations in the next analysis.

### Hypocenter locations

We used Hypoinverse version 1.40^31^ to determine hypocenter locations. This program determines earthquake locations by minimizing the misfit between observed and calculated travel times. Hypoinverse calculates earthquake depths from both the surface and the geoid (sea level). The flat earth model in the program assumes that earthquake depths are relative to the average local surface defined by nearby seismic stations^31^. We selected events that exceeded a reliability threshold based on a C rating in Hypoinverse, in which RMS < 0.5 s, azimuthal gap ≤ 180°, NWR ≥ 6, DMIN ≤ 50 km. ERH and ERZ are simplified errors that are based on the lengths and directions of the main axes of the error ellipsoid. Additionally, we set ERH < 2 km, and ERZ < 2 km as estimated hypocenters have a confidence interval of 95% if ERH and ERZ are ± 2.2 and 2 km, respectively^55^. The azimuthal gap, a proxy for station coverage, determines the accuracy of the epicenter location. The location accuracy decreases notably if azimuthal gap > 180°, representing one-sided coverage^56–58^.

We used double difference earthquake localization implemented in the HypoDD program^32^, including singular value decomposition, to refine the precision of the hypocenters. HypoDD optimally relocates seismic events in the presence of measurement errors and uncertainty in the velocity model. We adjusted hypocenter depths relative to sea level by subtracting the hypocenter depths from the output of HypoDD with average elevation of five nearby stations^31^. Note that our results were based on a 1D velocity model. Future studies should consider using an updated 3D velocity model to improve results.

### Ethics declarations

The authors declare that they have no known competing financial interests or personal relationships that could have appeared to influence the work reported in this paper.

## Supplementary Information

Supplementary Figures.

## Data Availability

The data that support the findings of this study are available from the corresponding author upon reasonable request. GSMaP precipitation data can be accessed through JAXA’s EORC portal (https://sharaku.eorc.jaxa.jp/GSMaP/).
