# Supplementary material for: Tracking supercritical geothermal fluid distribution from continuous seismic monitoring
Source: Sci Rep. 2023 May 24;13:8370. doi: 10.1038/s41598-023-35159-8 (PMC10209096; doi:10.1038/s41598-023-35159-8)
Supplement: Supplementary file 1 — Supplementary Figures. [file 41598_2023_35159_MOESM1_ESM.docx]

**Supplementary information for:**

**Tracking supercritical geothermal fluid distribution from continuous seismic monitoring**


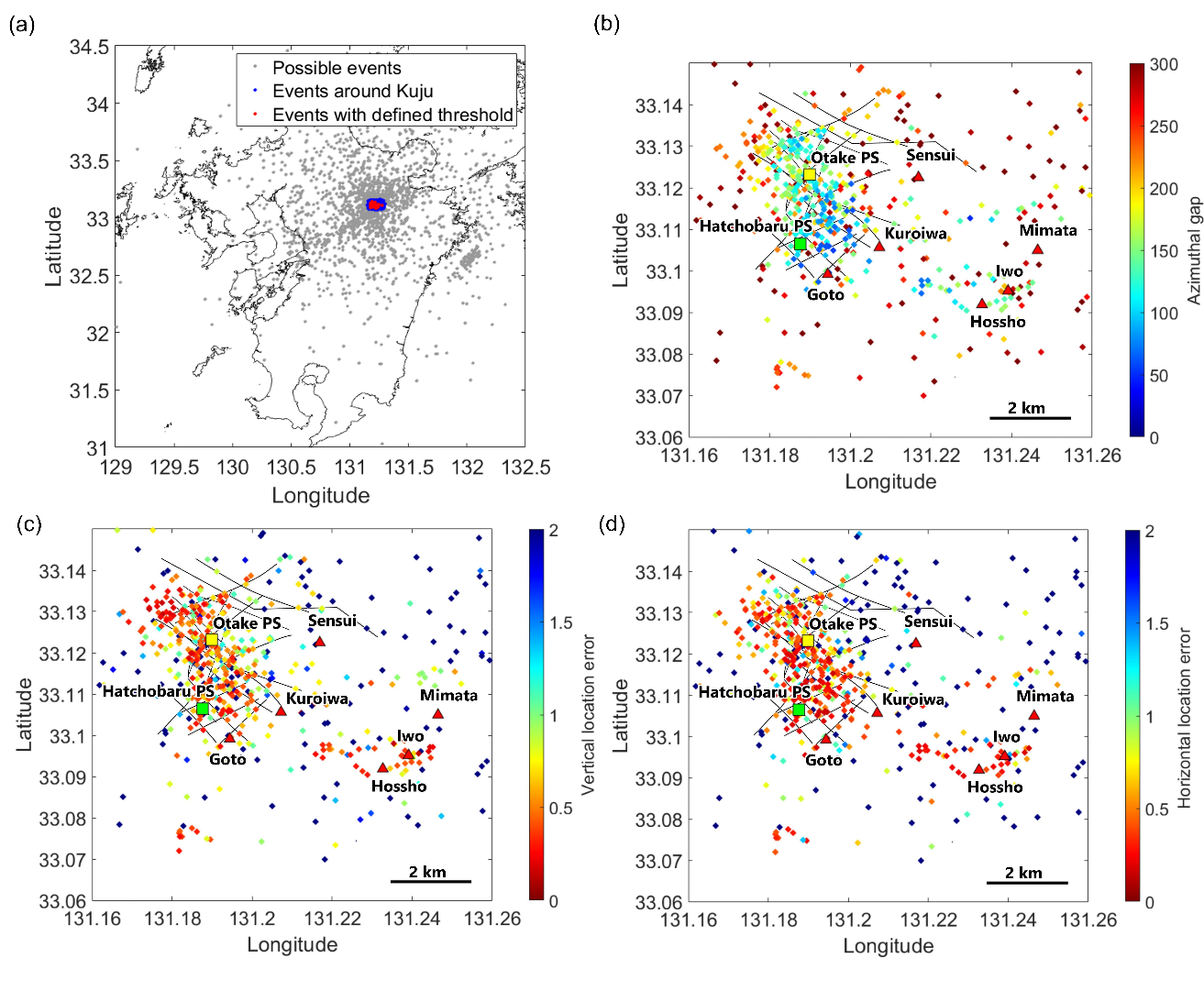


**Supplementary Figure S1.** Parameter of azimuthal gap, vertical error, and horizontal error which contribute the most to the events selection**.** (a) Plan view of the events. The gray color represents all possible events from phase association (~2600 events). The blue dots mark the seismicity close to our seismic network, the red dots mark the seismicity defined by the threshold in this manuscript. The Hypoinverse output for (b) azimuthal gap, (c) vertical location error (ERZ), and (d) horizontal location error (ERH) for each seismometer’s location.


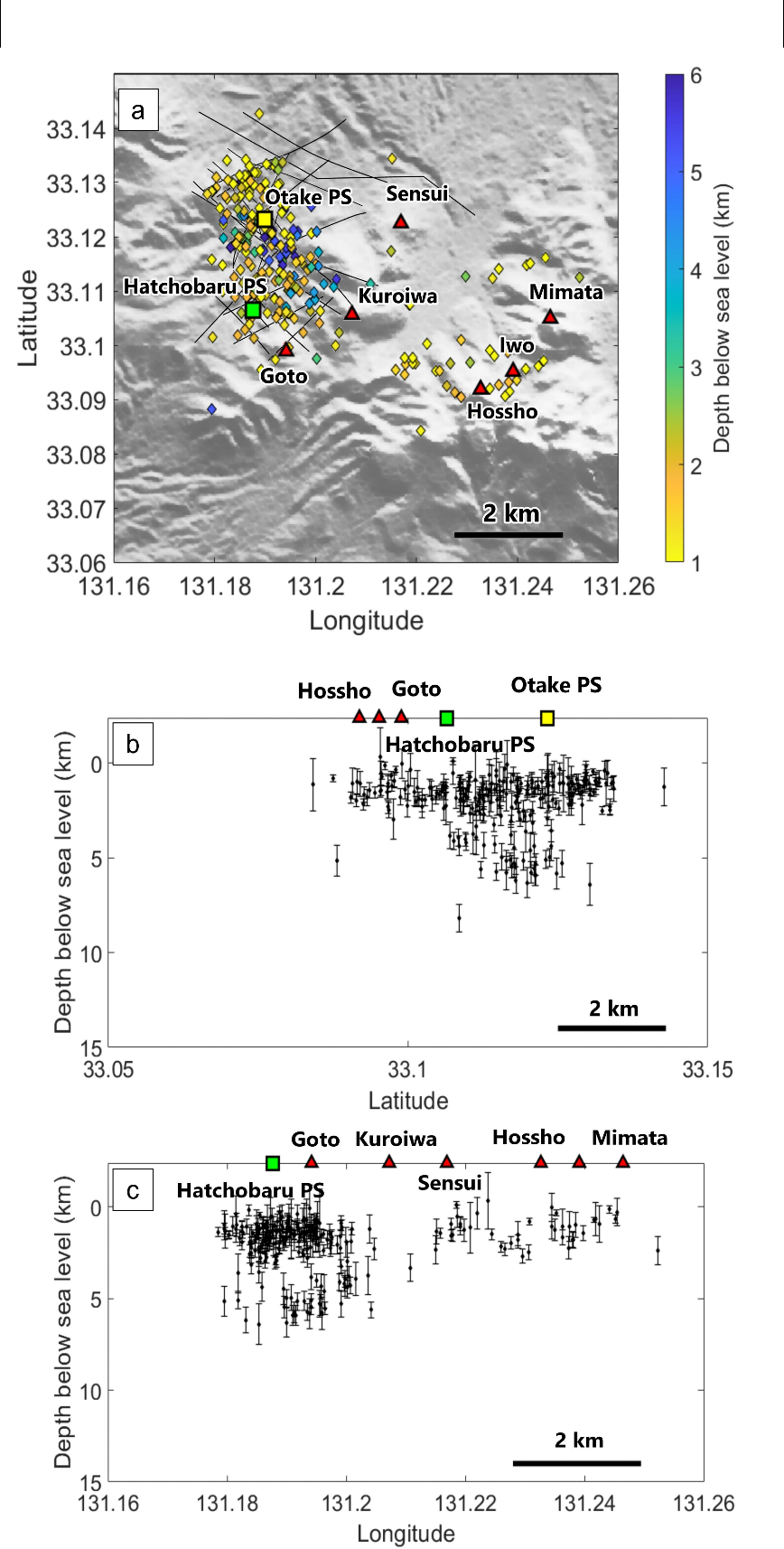


**Supplementary Figure S2.** Hypocenter results from Hypoinverse**.** (a) Plan view of the events. Panel (b) and (c) show the seismicity distribution in vertical view. The error bar represents the length of the vertical location error.


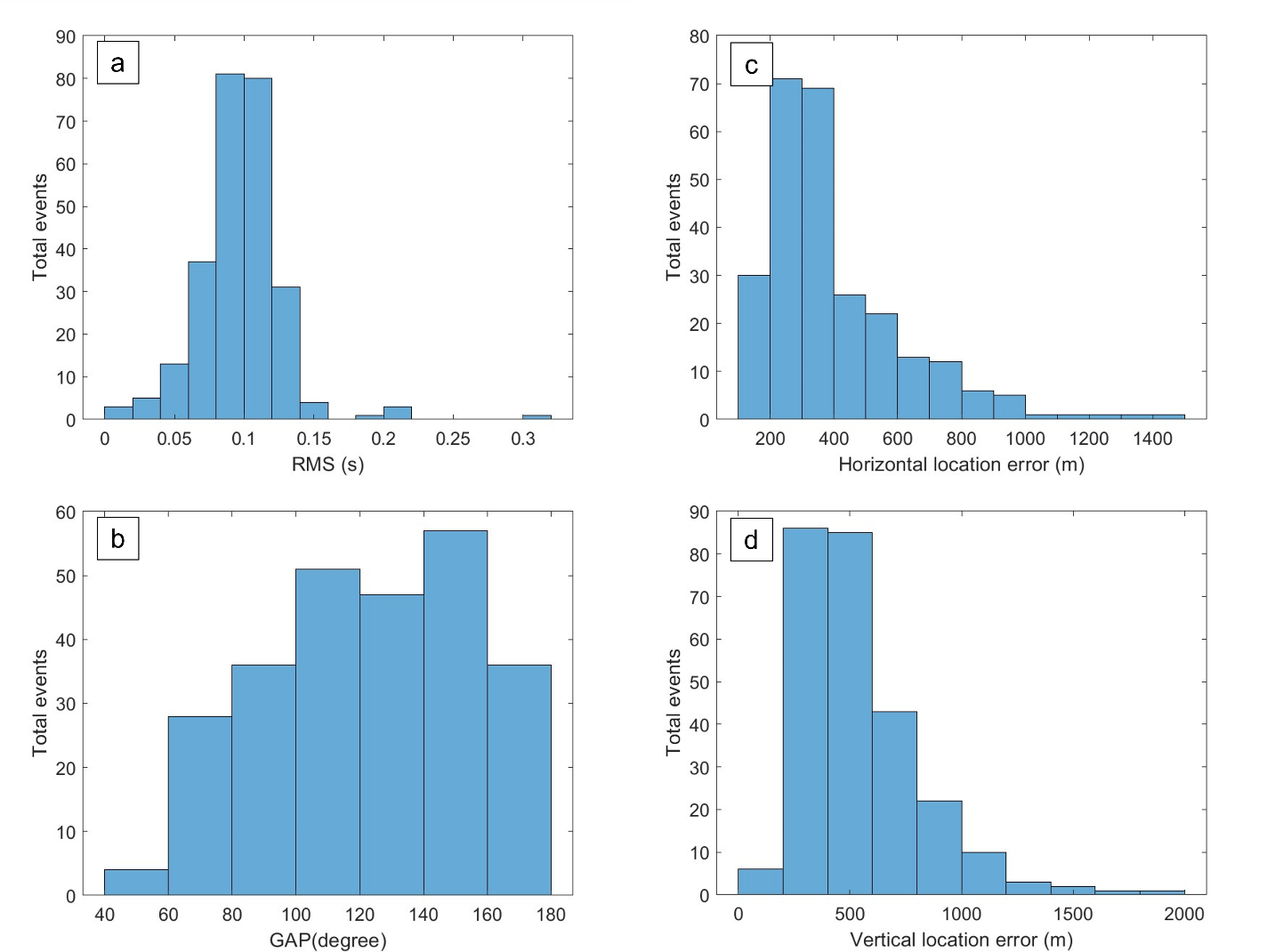


**Supplementary Figure S3.** Hypocenter quality output from Hypoinverse. Histogram of (a-d) RMS, azimuthal gap, horizontal location error, and vertical location error, respectively.
